# RISK FACTORS AT NON-UNION OF TIBIAL FRACTURE TREATED WITH INTRAMEDULLARY NAIL

**DOI:** 10.1590/1413-785220243202e278581

**Published:** 2024-06-24

**Authors:** Vitor Lorens Yulta Abe Puccetti, Fernando Loureiro de Miranda, Caio Cesar Nogueira de Figueiredo, Kayo Augusto de Almeida Medeiros, Marcos de Camargo Leonhardt, Jorge dos Santos Silva, Kodi Edson Kojima

**Affiliations:** 1 Universidade de São Paulo, Medical School, Hospital das Clínicas (HC-FMUSP), Institute of Orthopedics and Traumatology, São Paulo, SP, Brazil.

**Keywords:** Tibial Fracture, Fracture Healing, Fractures, Ununited, Fraturas da Tíbia, Consolidação da Fratura, Fraturas não Consolidadas

## Abstract

**Objective:**

Identify the predictors associated with delayed union at 6 months and non-union at 12 months in tibial shaft fractures treated with intramedullary nailing (IMN).

**Methods:**

This retrospective longitudinal study included a cohort of 218 patients who sustained tibial shaft fractures and received IMN between January 2015 and March 2022. We gathered data on a range of risk factors, including patient demographics, trauma intensity, associated injuries, fracture characteristics, soft tissue injuries, comorbidities, addictions, and treatment-specific factors. We employed logistic bivariate regression analysis to explore the factors predictive of delayed union and non-union.

**Results:**

At the 6-month follow-up, the incidence of delayed union was 28.9%. Predictors for delayed union included flap coverage, high-energy trauma, open fractures, the use of external fixation as a staged treatment, the percentage of cortical contact in simple type fractures, RUST score, and postoperative infection. After 12 months, the non-union rate was 15.6%.

**Conclusion:**

the main predictors for non-union after IMN of tibial shaft fractures are related to the trauma energy. Furthermore, the initial treatment involving external fixation and postoperative infection also correlated with non-union. ***Level of Evidence III; Retrospective Longitudinal Study.***

## RESUMO

**Objetivo:**

identificar os fatores preditivos associados ao atraso de consolidação em 6 meses e à não união em 12 meses em fraturas da diáfise da tíbia tratadas com haste intramedular (HIM).

**Métodos:**

O estudo longitudinal retrospectivo de coorte incluiu 218 pacientes, que apresentaram fraturas da díafise da tíbia e receberam HIM entre janeiro de 2015 e março de 2022. Os desfechos principais pesquisados foram atraso de consolidação em 6 meses de acompanhamento, e não união em 12 meses. Coletou-se dados de uma variedade de fatores de risco. Utilizou-se análise de regressão logística bivariada para explorar os fatores preditivos de atraso de consolidação e não união.

**Resultados:**

Aos 6 meses, a incidência de atraso de consolidação foi de 28,9%. Os preditores de atraso de consolidação incluem cobertura de retalho, trauma de alta energia, fraturas expostas, uso de fixação externa como tratamento estagiado, porcentagem de contato cortical em fraturas simples, escore RUST e infecção pós-operatória. Após 12 meses, a taxa de não união foi de 15,6%, com fatores preditivos sendo necessidade de cobertura por retalho, lesão vascular, trauma de alta energia, fraturas expostas, uso de fixação externa como tratamento estagiado, porcentagem de contato cortical em fraturas simples e infecção pós-operatória. ***Nível de Evidência III; Estudo Longitudinal Retrospectivo.***

## INTRODUCTION

Tibial shaft fractures are the most prevalent type of long bone fracture, demonstrating a bimodal distribution. Intramedullary nailing (IMN) stands as the primary treatment for displaced tibial shaft fractures^2^. Despite its effectiveness, complications such as delayed union and non-union continue to pose a substantial challenge, with reported incidence rates ranging from 4% to 48%^3,4^.

The consequences of delayed union and non-union extend beyond statistics. These complications impose additional burden on patients, necessitating revision surgeries and prolonging pain and disability.

Numerous previous studies have endeavored to shed light on the factors influencing non-union development, including patient demographics, injury and fracture characteristics, and aspects related to treatment^5,6^. However, the current body of literature remains marked by uncertainties and inconclusive findings regarding the precise risk factors for fracture healing disturbances^7,8^.

The primary objectives of this study are to elucidate the risk factors and predictors associated with delayed union at 6 months and non-union at 12 months following the intramedullary nailing treatment of tibial shaft fracture.

## METHODS

This retrospective study was conducted at urban university-based level one trauma center. Data were collected through a retrospective chart review and the review of existing radiographs from patients with tibial shaft fractures who underwent fixation with IMN, between January 2015 and March 2022. Ethical approval was granted by the Scientific and Ethical Committee (SEC) of the University under the protocol number 24061. Given the retrospective nature of the study, a request was submitted to the SEC to waive the need for the informed consent from the patients, and it was approved.

The inclusion criteria were as follows: age over 18 years, fracture of the tibia shaft, closed or open, treated with intramedullary nailing, follow up radiographs at six months and 12 months, and availability of all necessary data in the patient's charts.

The exclusion criteria included pathologic fractures, proximal or distal fractures of the tibia, diaphyseal bone loss, prior injury to the same tibia, and treatments other than IMN.

Data were collected on patient's preoperative, intraoperative, and postoperative information. All the relevant data potentially influencing the healing process were collected. These factors were considered to establish their association as risk factor for delayed or non-union. Patient characteristics: age, sex, and race.

Trauma energy: high-energy (e.g., car accidents, firearm injuries, fall from height, motorbike accident and vehicle collision) and low energy (e.g., fall from standing height, sports injuries, blunt trauma). Associated injuries: chest and abdominal injuries, neurovascular damage, and fractures in other segments. These injuries were classified according to the Abbreviated Injury Scale (AIS), and subclassified into AIS ≤ 2 and AIS ≥ 3^9^.

Fracture characteristics: side, shaft segment (proximal, middle, distal), AO/OTA (AO Foundation / Orthopedic Trauma Association) classification^10^, open fractures (Gustilo classification)^11^, in AO/OTA simple type fractures the percentage of cortical contact (< 25%, 25% to 50%, 50% to 75%, > 75% and 100%), for the type B fractures more or less than 50% of contact.

Soft tissue injury: flap reconstruction, vascular injury and compartment syndrome.

Comorbidities and addictions: obesity, diabetes mellitus, and any other relevant comorbidity, as well as smoking, alcoholism, use of illicit drugs.

Treatment: treatment with external fixator, time to conversion into IMN, time between the fracture and the IMN fixation in non-staged cases, reaming or not reaming, gap > 2 mm between the fragments. Radiographic evaluation: Radiographic Union Scale in tibial fracture (RUST) was used to assess the bone healing. A score of 1 indicated no callus, 2 indicated initial callus with the fracture line still visible, 3 indicated callus with no fracture line visible. It was based on the sum of the four cortical scores (two in the anteroposterior and two in the lateral view)^12^.

Healing: fracture was considered healed when patients had no pain in the fracture site, no limping and showed callus involving at least three of the four cortices and required additional surgical intervention beyond definitive fixation^13^. Lack of healing in 6 months was classified as delayed union and absence of healing at 12 months was classified as non-union.

Follow up: assessment of screw breakage and deep infection.

Qualitative parameters assessed were described for all patients using absolute and relative frequencies and the qualitative characteristics were described using summary measures (mean and standard deviation). The occurrence of delayed union at 6 months and non-union at 12 months was described according to the qualitative characteristics using absolute and relative frequencies and the association was verified using chi-square tests or exact test (Fischer's exact test or likelihood ratio tests). The quantitative characteristics were described according to each outcome using summary measures and compared using Student's t-test. Unadjusted odds ratios (OR) were estimated with the respective 95% confidence intervals for each variable of interest for non-union in each period using bivariate logistic regression and joint models were created with the characteristics that had a descriptive level of less than 0.10 (p < 0.10) in the unadjusted analyzes, with the characteristics being present for all the patients in the study and whose numbers of patients in the categories were in agreement to be included in the analyzes, with the models being carried out using multiple logistic regression with full models, i.e., all the variables included in the models were kept in the final models^14,15^.

The IBM-SPSS for Windows version 22.0 software was used to carry out the analysis and Microsoft Excel 2013 was used to tabulate the data and create the graphs. The tests were carried out at a 5% significance level.

## RESULTS

From January 2015 to March 2022, our cohort encompassed a total of 218 patients. The cohort exhibited a mean age of 36.2 ± 14.2 years, with a male predominance comprising 180 patients (82.6%). High-energy trauma constituted the etiological factor in 84.9% of the cases, and 50.5% of these cases presented with associated injuries, of which 52.7% were classified as AIS >3. (Table 1)

**Table 1:** Demographic data.

Variable	Description (n = 218)
Age (years), mean SD	36.2 ± 14.2
Sex (male), n (%)	180 (82.6)
Race (white), n (%)	172 (78.9)
High energy trauma, n (%)	185 (84.9)
**Associated injuries, n (%)**	
No	108 (49.5%)
AIS < 3	58 (26.6)
AIS ≥ 3	52 (23.9)
**AO/OTA classification, n (%)**	
Type A	125 (57.3)
Type B	55 (25.2)
Type C	38 (17.4)
**Gustilo classification, n (%)**	
Closed	80 (36.7)
Type I	4 (1.8)
Type II	2 (0.9)
Type IIIA	107 (49.1)
Type IIIB	20 (9.2)
Type IIIC	5 (2.3)
**Side, n (%)**	
Right	92 (42.2)
Left	123 (56.4)
Bilateral	3 (1.4)
**Shaft segment, n (%)**	
Proximal	10 (4.6)
Middle	129 (59.2)
Distal	79 (36.2)
Obesity, n (%)	3 (1.4)
Smokers, n (%)	23 (10.6)
Alcoholism, n (%)	11 (5)
Illicit drugs, n (%)	12 (5.5)
Diabetes, n (%)	6 (2.8)
Other comorbidities, n (%)	24 (11)

n = number, SD = standard deviation.

The prevailing fracture type was the AO/OTA type A, accounting for 57.3% of the cases. The majority of the fractures were characterized as open injuries (63.3%), with 49.1% classified as Gustilo IIIA and 9.2% as Gustilo IIIB. Compartment syndrome occurred in only 5 (2.3%) cases. (Table 1)

For a more comprehensive dataset of the patients’ characteristics, please refer to Table 1.

The average interval between fracture occurrence and IMN fixation was 8.5 ± 5.7 days. Among the patients who underwent the staged treatment with the external fixator 135 (61.9), the average time to conversion into IMN was 6.1 ± 6.1 days. Reaming was performed in 146 (67%) cases. (Table 1)

A fracture gap greater than 2 mm was observed in 132 cases (60.6%) following IMN. The final reduction revealed less than 50% contact in 61% of type A fractures and 41.8% of type B fractures. (Table 2) Regarding the radiographic assessment of the healing process, the RUST at the 6-month follow up was 8.2 ± 2.2. Fracture healing was observed in 155 patients (71.1%) at the 6-month mark, increasing to 184 patients (84.4%) at the 12-month follow up leading to a non-union rate of 15.6%. (Table 2)

**Table 2:** Results related to the treatment.

Variable	Description (n = 218)
Flap reconstruction, n (%)	29 (13.3)
Vascular injury, n (%)	5 (2.3)
Compartment syndrome, n (%)	5 (2.3)
Staged external fixator, n (%)	135 (61.9)
Days to convert, mean SD	6.1 6.1
Days to definitive IMN, mean SD	8.5 5.7
Gap > 2 mm, n (%)	132 (60.6)
**Type A cortical contact, n (%)**	
< 25%	44 (35.2)
25% - 50%	32 (25.6)
50 - 75%	37 (29.6)
100%	12 (9.6)
**Type B cortical contact, n (%)**	
< 50%	23 (41.8)
> 50%	37 (29.6)
Reamed nail, n (%)	146 (67)
RUST 6m, mean SD	8.2 2.2
Healed 6 months, n (%)	155 (71.1)
Healed 12 months, n (%)	184 (84.4)
**Locking bolt breakage, n (%)**	
No	208 (95.4)
Proximal	6 (2.8)
Distal	4 (1.8)
Deep infection, n (%)	29 (13.3)

n = number, SD = standard deviation.

Locking bolt breakage occurred in 10 patients (4.6%) and deep infection emerged as complication in 29 patients (13.3%). (Table 2) At the six-month follow-up evaluation, several factors exhibited statistically significant correlations with delayed union. These included the need for flap reconstruction (p < 0.001), high-energy trauma (p < 0.001), open fractures (p < 0.001), staged treatment involving initial external fixation (p < 0.001), the number of days to convert the external fixator to IMN (p = 0.006), cortical contact in type A fractures less than 50% (p < 0.001), RUST (p < 0.001), and deep infection (p < 0.001). (Table 3)

**Table 3:** Statistical analyzes of healing with 6-month follow up.

Variable	Healing in 6 m		OR	IC (95%)		p
	Yes	No		Inferior	Superior	
Age (years), mean SD	36,8 14,6	34,8 13,2	0,99	0,97	1,01	
Sex						0,117
Female	31 (81,6)	7 (18,4)	1,00			
Male	124 (68,9)	56 (31,1)	2,00	0,83	4,81	
Race						0,765
White	122 (70,9)	50 (29,1)	1,00			
Brown	19 (67,9)	9 (32,1)	1,16	0,49	2,72	
Black	14 (77,8)	4 (22,2)	0,70	0,22	2,22	
Flap reconstruction						>0,001
No	143 (75,7)	46 (24,3)	1,00			
Yes	12 (41,4)	17 (58,6)	4,41	1,96	9,90	
Vascular injury						0,146*
No	153 (71,8)	60 (28,2)	1,00			
Yes	2.140]	3 (60)	3,83	0,62	23,26	
Compartment syndrome						0,628*
No	152 (71,4)	61 (28,6)	1,00			
Yes	3 (60)	2 (40)	1,66	0,27	10,20	
Obesity						>0,999*
No	153 (71,2)	62 (28,8)	1,00			
Yes	2 (66,7)	1 (33,3)	1,23	0,11	13,89	
Smoker						0,753
No	138 (70,8)	57 (29,2)	1,00			
Yes	17 (73,9)	6 (26,1)	0,85	0,32	2,28	
Alcoholism						0,517*
No	146 (70,5)	61 (29,5)	1,00			
Yes	9 (81,8)	2 (18,2)	0,53	0,11	2,53	
Illicit drugs						>0,999*
No	146 (70,9)	60 (29,1)	1,00			
Yes	9 (75)	3 (25)	0,81	0,21	3,10	
Diabetes						>0,999*
No	151 (71,2)	61 (28,8)	1,00			
Yes	4 (66,7)	2 (33,3)	1,24	0,22	6,94	
Other comorbidities						0,324
No	140 (72,2)	54 (27,8)	1,00			
Yes	15 (62,5)	9 (37,5)	1,56	0,64	3,76	
Trauma energy						>0,001
Low	33 (100)	0 (0)	1,00			
High	122 (65,9)	63 (34,1)	&			
Associated injury						0,179
No	83 (76,9)	25 (23,1)	1,00			
AIS < 3	38 (65,5)	20 (34,5)	1,75	0,87	3,52	
AIS ≥ 3	34 (65,4)	18 (34,6)	1,76	0,85	3,64	
Gustilo						<0,001#
Closed	64 (80)	16 (20)	1,00			
I	4 (100)	0 (0)	&			
II	2 (100)	0 (0)	&			
IIIA	78 (72,9)	29 (27,1)	1,49	0,74	2,98	
IIIB	5 (25)	15 (75)	12,00	3,80	37,93	
IIIC	2 (40)	3 (60)	6,00	0,92	38,98	
Side						0,356#
Right	65 (70,7)	27 (29,3)	1,00			
Left	87 (70,7)	36 (29,3)	1,00	0,55	1,80	
Bilateral	3 (100)	0 (0)	&			
Bilateral						0,558*
No	152 (70,7)	63 (29,3)	1,00			
Yes	3 (100)	0 (0)	&			
Segment in the shaft						0,056#
Proximal	4 (40)	6 (60)	1,00			
Middle	90 (69,8)	39 (30,2)	0,29	0,08	1,08	
Distal	61 (77,2)	18 (22,8)	0,20	0,05	0,77	
Staged with external fixator, n (%)						<0,001
No	73 (88)	10 (12)	1,00			
Yes	82 (60,7)	53 (39,3)	4,72	2,24	9,90	
Reamed IMN						0,099
No	46 (63,9)	26 (36,1)	1,00			
Yes	109 (74,7)	37 (25,3)	0,60	0,33	1,10	
Days to convert to IMN, mean SD	5,4 6,2	7,9 5,6	1,07	1,02	1,12	0,006**
Days to IMN, mean SD	8,4 5,7	8,9 5,5	1,02	0,97	1,07	0,488**
Gap between fragments						0,383
< 2mm	64 (74,4)	22 (25,6)	1,00			
> 2mm	91 (68,9)	41 (31,1)	1,31	0,71	2,41	
AO/OTA classification						0,120
A	94 (75,2)	31 (24,8)	1,00			
B	39 (70,9)	16 (29,1)	1,24	0,61	2,53	
C	22 (57,9)	16 (42,1)	2,21	1,03	4,72	
Type A cortical contact						<0,001#
< 25%	23 (52,3)	21 (47,7)	4,57	0,90	23,29	
25% - 50%	26 (81,3)	6 (18,8)	1,15	0,20	6,70	
50% - 75%	35 (94,6)	2 (5,4)	0,29	0,04	2,29	
100%	10 (83,3)	2 (16,7)	1,00			
Type B cortical contact						0,431
< 50%	15 (65,2)	8 (34,8)	1,60	0,50	5,17	
> 50%	24 (75)	8 (25)	1,00			
RUST 6 m, mean SD	9,3 1,4	5,6 1,5	0,21	0,14	0,33	<0,001**
Locking bolt breakage						0,081#
No	151 (72,6)	57 (27,4)	1,00			
Yes (Proximal)	3 (50)	3 (50)	2,65	0,52	13,51	
Yes (Distal)	1 (25)	3 (75)	7,94	0,81	76,92	
Deep infection						<0,001
No	143 (75,7)	46 (24,3)	1,00			
Yes	12 (41,4)	17 (58,6)	4,41	1,96	9,90	

Chi-square test;

* Fisher exact test;

# Likelihood ratio test;

** unpaired Student t test; & Not enough case to estimate.

In the 12-month follow-up assessment, factors that remained statistically significant in their correlation with non-union included the need for flap reconstruction (p = 0.001), high-energy trauma (p = 0.007), open fractures (p < 0.001), staged treatment involving an external fixator (p = 0.003), cortical contact less than 50% in type A fractures (p < 0.001), RUST (p < 0.001), and deep infection (p = 0.002). Additionally, vascular injury showed correlation (p = 0.029) in this group. (Table 4) Multiple logistic regression analyses encompassing all risk factors revealed that, at the 6-month mark, patients who used an external fixator had a 5.99 times higher chance of experiencing delayed union compared to those who did not use one (p = 0.016). Furthermore, with each 1-point increase in RUST, the chance of delayed union decreased by 79% (p < 0.001), irrespective of other patient characteristics. Patients requiring flap reconstruction had a 2.99 times higher chance of non-union at 12 months compared to those without the need for a flap (p = 0.027). Patients subjected to prior external fixation had a 4-fold higher chance of non-union at 12 months compared to those who did not undergo external fixation (p = 0.031). Lastly, patients with postoperative deep infections had a 2.87 times higher chance of experiencing non-union, regardless of other patient characteristics. (Table 5)

**Table 4:** Statistical analyzes of healing with 12 months follow up.

Variable	Healing in 6 m		OR	IC (95%)		p
	Yes	No		Inferior	Superior	
Age (years), mean SD	36,8 14,3	34,8 14,7	0,99	0,96	1,02	0,462**
Sex						0,058
Female	35 (94,6)	2 (5,4)	1,00			
Male	142 (82,1)	31 (17,9)	3,82	0,87	16,67	
Race						0,571#
White	136 (82,9)	28 (17,1)	1,00			
Brown	25 (89,3)	3 (10,7)	0,58	0,16	2,07	
Black	16 (88,9)	2 (11,1)	0,61	0,13	2,79	
Flap reconstruction						0,001*
No	161 (88)	22 (12)	1,00			
Yes	16 (59,3)	11 (40,7)	5,03	2,07	12,20	
Vascular injury						0,029*
No	175 (85,4)	30 (14,6)	1,00			
Yes	2 (40)	3 (60)	8,77	1,40	55,56	
Compartment syndrome						0,578*
No	173 (84,4)	32 (15,6)	1,00			
Yes	4 (80)	1 (20)	1,35	0,15	12,50	
Obesity						>0,999*
No	174 (84,1)	33 (15,9)	1,00			
Yes	3 (100)	0 (0)	&			
Smoker						>0,999*
No	159 (84,1)	30 (15,9)	1,00			
Yes	18 (85,7)	3 (14,3)	0,88	0,24	3,18	
Alcoholism						>0,999*
No	167 (83,9)	32 (16,1)	1,00			
Yes	10 (90,9)	1 (9,1)	0,52	0,06	4,22	
Illicit drugs						>0,999*
No	167 (83,9)	32 (16,1)	1,00			
Yes	10 (90,9)	1 (9,1)	0,52	0,06	4,22	
Diabetes						>0,999*
No	172 (84,3)	32 (15,7)	1,00			
Yes	5 (83,3)	1 (16,7)	1,08	0,12	9,52	
Other comorbidities						0,549*
No	158 (84,9)	28 (15,1)	1,00			
Yes	19 (79,2)	5 (20,8)	1,49	0,51	4,31	
Trauma energy						0,007
Low	33 (100)	0 (0)	1,00			
High	144 (81,4)	33 (18,6)	&			
Associated injury						0,428
No	91 (87,5)	13 (12,5)	1,00			
AIS < 3	46 (82,1)	10 (17,9)	1,52	0,62	3,73	
AIS ≥ 3	40 (80)	10 (20)	1,75	0,71	4,33	
Gustilo						<0,001#
Closed	69 (88,5)	9 (11,5)	1,00			
I	4 (100)	0 (0)	&			
II	2 (100)	0 (0)	&			
IIIA	91 (89,2)	11 (10,8)	0,93	0,36	2,36	
IIIB	9 (47,4)	10 (52,6)	8,52	2,73	26,56	
IIIC	2 (40)	3 (60)	11,50	1,69	78,39	
Side						0,596#
Right	74 (84,1)	14 (15,9)	1,00			
Left	100 (84)	19 (16)	1,00	0,47	2,13	
Bilateral	3 (100)	0 (0)	&			
Bilateral						>0,999*
No	174 (84,1)	33 (15,9)	1,00			
Yes	3 (100)	0 (0)	&			
Segment in the shaft						0,252#
Proximal	6 (75)	2 (25)	1,00			
Middle	103 (81,7)	23 (18,3)	0,67	0,13	3,53	
Distal	68 (89,5)	8 (10,5)	0,35	0,06	2,05	
Staged with external fixator, n (%)						0,003
No	76 (93,8)	5 (6,2)	1,00			
Yes	101 (78,3)	28 (21,7)	4,22	1,56	11,36	
Reamed IMN						0,899
No	57 (83,8)	11 (16,2)	1,00			
Yes	120 (84,5)	22 (15,5)	0,95	0,43	2,09	
Days to convert to IMN, mean SD	5,8 6,2	7,7 5,9	1,05	0,99	1,11	0,105**
Days to IMN, mean SD	8,5 5,7	9 5,8	1,02	0,95	1,08	0,613**
Gap between fragments						0,238
< 2mm	73 (88)	10 (12)	1,00			
> 2mm	104 (81,9)	23 (18,1)	1,62	0,73	3,60	
AO/OTA classification						0,067
A	106 (87,6)	15 (12,4)	1,00			
B	46 (85,2)	8 (14,8)	1,23	0,49	3,10	
C	25 (71,4)	10 (28,6)	2,82	1,14	7,04	
Type A cortical contact						<0,001#
< 25%	31 (72,1)	12 (27,9)	&			
25% - 50%	28 (90,3)	3 (9,7)	&			
50% - 75%	36 (100)	0 (0)	&			
100%	11 (100)	0 (0)	1,00			
Type B cortical contact						>0,999*
< 50%	19 (86,4)	3 (13,6)	0,85	0,18	4,01	
> 50%	27 (84,4)	5 (15,6)	1,00			
RUST 6 m, mean SD	8,9 1,7	5,2 1,3	0,33	0,23	0,46	<0,001**
Locking bolt breakage						0,142#
No	171 (85,5)	29 (14,5)	1,00			
Yes (Proximal)	4 (66,7)	2 (33,3)	2,95	0,52	16,95	
Yes (Distal)	2 (50)	2 (50)	5,88	0,80	43,48	
Deep infection						0,002*
No	161 (87,5)	23 (12,5)	1,00			
Yes	16 (61,5)	10 (38,5)	4,37	1,77	10,75	

Chi-square test;

* Fisher exact test;

# Likelihood ratio test;

** unpaired Student t test; & Not enough case to estimate.

**Table 5:** Multiple regression logistic analyzes of the healing in 6 and 12 months and the risk factors.

Outcome	Risk factor	OR	IC (95%)		p
			Inferior	Superior	
Healing in 6 months	Flap reconstruction	0,85	0,21	3,46	0,817
	Shaft segment				
	Proximal (ref.)	1,00			
	Middle	0,23	0,02	3,15	0,273
	Distal	0,41	0,03	5,65	0,506
	External fixator	5,99	1,40	25,64	0,016
	Reamed IMN	0,30	0,09	1,06	0,061
	RUST 6 months	0,21	0,13	0,33	<0,001
	Deep infection	4,15	0,88	19,61	0,071
Healing in 12 months	Flap reconstruction	2,99	1,14	7,94	0,027
	Vascular injury	4,00	0,49	32,26	0,194
	External fixator	3,12	1,11	8,77	0,031
	Deep infection	2,87	1,08	7,63	0,034

Multiple regression logistic analyzes.

## DISCUSSION

Non-union, a distressing complication, may ensue after a fracture, imposing considerable physical and economic burdens. This phenomenon not only inflicts substantial pain, discomfort and functional impairment to the patient but also necessitates additional medical interventions, incurring in substantial expenses^13,16^.

The importance of this issue is further exacerbated when it pertains to non-union arising from tibia shaft fractures, given their status as the most prevalent long bone fractures in adults^1,2^, thereby amplifying the magnitude of the problem.

This is the first to study a population in Brazil and Latin America with a substantial sample size. Notably, the average age of our patient cohort stood at 36.2 ± 14.2, signifying a youthfulness in comparison to analogous studies such as Kawasaki N et al.^4^ and Makaram NS et al.^17^, which reported mean ages of 45.6 and 46 years, respectively. This deviation may be explained to the unique characteristics of our institution - a tertiary trauma center entrusted with the most severe cases within the city's rescue system.

Given the prominence of high-energy trauma, one might anticipate a concomitant prevalence of associated injuries. However, our study diverges from this expectation, revealing that nearly half of our patients (49.5%) presented without any associated injuries. Among those who did, the injuries tended to be minor in nature (AIS < 3). This phenomenon can be explained by the preponderance of motorcycle accidents within our city. Such incidents frequently result in extremity injuries while sparing the abdomen or thorax from trauma, thus accounting for this distribution of injury pattern. Among the 138 patients in our study, representing 63.3% of the total cohort, 107 patients (77.5%) presented with Gustilo IIIA lesions, while 20 patients (14.5%) exhibited type IIIB lesions, necessitating attention to soft tissue reconstruction with flap coverage. However, it is noteworthy that 29 patients underwent flap reconstruction, that is explained by the fact that nine patients from the Gustilo IIIA group encountered postoperative soft tissue complications, requiring debridement and subsequent soft tissue reconstruction. Both open fracture and need for flap had association with the incidence of delayed union at 6 months of 28.9% and non-union at 12 months of 15.6% ((p < 0.001).

Despite the predominance of high-energy mechanism as the primary etiological factor, the incidence of vascular injuries was relatively low, observed in only five patients (2.3%). A similar trend was noted for compartment syndrome, affecting only five patients (2.3%).

On average, fractures that did not necessitated staged treatment with external fixation were stabilized using IMN approximately 8.5 ± 5.7 days post-fracture. Importantly, this delay in fixation did not exhibit any significant association with disturbance in the healing process (p = 0.488).

The staged treatment protocol was indicated for 135 patients, comprising 61.9% of our study cohort. Notably, prior use of external fixation demonstrated a strong association with both delayed and non-union outcomes (p < 0.001). This phenomenon can be attributed to the specific indication for external fixation, which is typically reserved for patients with systemic compromise, like polytrauma, or severe soft tissue injuries. Both these factors are known to significantly influence the healing process, potentially delaying, or impeding it.

Interestingly, the time to conversion to the IMN, with an average of 6.1 ± 6.1 days, did not exhibit a significant association with disruption in the healing process.

In our research, the utilization of reaming or on-reaming procedures exhibited no statistically significant association with non-union incidence (p = 0.899). The debate surrounding the advantages of reamed nail insertion in the context of fracture healing remains ongoing. A comprehensive systematic review conducted by Clark DR et al.^18^, which included six relevant studies, leans towards endorsing the use of reamed nails. However, it is worth noting that the overall quality of these studies falls within a moderate range. Conversely, Xia L et al.^19^, in their meta-analysis, suggest that reamed nailing may lower the risk of non-union in closed fractures, in a different perspective, Schemitsch EH et al.^20^ reported findings that indicate neither reaming nor non-reaming significantly affects reoperation rates. Notably, our series primarily includes open fractures, and this fact seems to align with the argument that reaming may not significantly impact open fracture outcome.

The RUST serves as valuable scoring system for assessing progress through radiographic imaging. Our study strongly supports the utility of RUST as a reliable predictor of delayed union at 6-month follow up. Remarkably, for each one-point increase in RUST score, there is in 79% reduction in the likelihood of delayed union (p < 0.001). To ensure the quality of our results, we deliberately excluded cases involving tibial shaft fractures with significant bone loss. It is self-evident that in absence of a contiguous cortical segment, fracture consolidation is unattainable without a reconstructive procedure. Our data underscores a observation: when cortical contact falls below 50% in simple type fractures, a significant association with non-union becomes evident (p < 0.001). however, in the case of B type fractures, proximal-to-distal segment contact does not exhibit a statistically significant association (p = 0.999). This discrepancy can likely be attributed to the overriding importance of the size and height of the wedge fragment in influencing the outcome.

In accordance with our expectations, a discernible correlation between postoperative deep infection and non-union has been established (p = 0.002). our observed infection incidence stands at 13.3%, and this is intrinsically linked to the substantial representation of patients afflicted with high-energy trauma and open fractures within our cohort.

Our study aligns with the findings of Ford et al.^21^, who reported a 27.9% non-union rate and an 11.5% incidence of deep infection. They identified high-energy trauma, open fractures, and early postoperative complications, including deep. Comorbidities play a diminishing role, whereas open fractures and staged external fixation become more critical.

Our study underscores that having less than 50% cortical contact is a significant non-union risk factor, corroborated by Bhandari et al^22^. and Fong et al^3^.

The clinical implications is, while these predictors are beyond a surgeon's control, they offer valuable insights for postoperative monitoring and intervention strategies. Although the choice of reaming has minimal impact, achieving a satisfactory reduction with more than 50% cortical contact is crucial, Furthermore, rigorous measures should be taken to prevent deep infection, as they strongly correlate with non-union risk.

## CONCLUSION

Our study identifies several key factors associated with heightened risk of non-union following IMN of tibial shaft fracture: high-energy trauma, open fracture, the need for flap procedures, staged external fixation treatment, less than 50% cortical contact, and deep infection.
